# Unraveling the virulence and host immune responses to *Enterococcus gallinarum* during a mass mortality event in bighead carp (*Hypophthalmichthys nobilis*)

**DOI:** 10.3389/fimmu.2026.1938548

**Published:** 2026-10-27

**Authors:** Vikash Kumar, Suvra Roy, Anupam Adhikari, Sneha Prakash, Sanjeev Kumar Singh, Asim Kumar Jana, Srikanta Samanta, Basanta Kumar Das

**Affiliations:** 1 Aquatic Environmental Biotechnology (AEB) Division, Indian Council of Agricultural Research (ICAR)-Central Inland Fisheries Research Institute (CIFRI), Barrackpore, India; 2 University Centre for Research & Development, Chandigarh University, Mohali, Punjab, India; 3 Fisheries Resource Assessment & Informatics (FRAI) Division, Indian Council of Agricultural Research (ICAR)-Central Inland Fisheries Research Institute (CIFRI), Barrackpore, India

**Keywords:** biofilm formation, East Kolkata Wetlands, innate immunity, MAR index, multidrug resistance, sewage-fed aquaculture, β-hemolysis

## Abstract

The pathogenicity of *Enterococcus gallinarum* in cultured fish is poorly characterized and emerging opportunistic bacteria pose an escalating threat to sustainable freshwater aquaculture. This study provides the first comprehensive phenotypic, histopathological and targeted candidate-gene expression analysis of *E. gallinarum* as an emerging multidrug-resistant (MDR) pathogen of bighead carp (*Hypophthalmichthys nobilis*) associated with a mass mortality event (~50% cumulative loss) in a sewage-fed system of the East Kolkata Wetlands, India. The disease was characterized by deep ulcerative lesions and cutaneous hemorrhage indicative of systemic infection. The clinical relevance of the dominant isolate is confirmed by robust identification using biochemical assays, 16S rRNA gene sequencing and phylogenetic analysis. Functional assays showed significant virulence, including biofilm formation greater than that of the *Aeromonas hydrophila* positive control, high swimming motility, and strong β-hemolysis. The *in vivo* pathogenic potential of the isolate was supported by 100% mortality following experimental challenge and recovery of *E. gallinarum* from internal tissues of challenged fish. The resistance to seven of 26 antimicrobials, including frontline drugs such as tetracyclines, macrolides, ansamycins, phenicols and carbapenems, and the high multiple antibiotic resistance (MAR) index of 0.34, suggest a significant therapeutic constraint and that antibiotics have been under constant selection pressure in the source environment. Histopathological examination showed widespread multi-organ injury, including hepatocellular necrosis, renal tubular necrosis, myofibrillar fragmentation, and splenic lymphoid depletion with melanomacrophage aggregates. Transcriptional profiling of brain, liver, kidney and muscle revealed a high but uncoordinated host response: *NF-κB* was uniformly up-regulated, *MHC II* was consistently down-regulated, *TLR4* and chemokine receptor transcripts were down-regulated in all tissues except muscle, the most responsive tissue, and *Hsp70, C3, IgM* and *NOD1* were regulated in a tissue-specific manner, suggesting that *E. gallinarum* not only uses host tissues but also interferes with pathogen recognition and antigen presentation. Taken together, these results outline the multifactorial nature of *E. gallinarum* virulence and MDR, highlighting its importance as an emerging pathogen in sewage-fed aquaculture. Future studies should focus on genomic dissection of its resistance and virulence determinants, longitudinal surveillance to understand transmission dynamics and the spread of AMR across the wetland aquaculture interface, and experimental testing of alternative mitigation strategies such as probiotics, phage therapy, and immune-modulating interventions to decrease the need for antibiotic use and support antimicrobial stewardship in sewage-fed aquaculture systems.

## Introduction

Aquaculture has become one of the fastest-growing food production sectors worldwide and plays a pivotal role in ensuring global food and nutritional security (1). However, the sustainability of intensive aquaculture is increasingly challenged by emerging infectious diseases that cause substantial economic losses through reduced growth, increased mortality, and compromised fish welfare. Among bacterial diseases, opportunistic pathogens have gained considerable attention because environmental deterioration, intensive farming practices, and climate-driven stressors enhance their pathogenic potential. Consequently, the emergence of previously overlooked bacterial species has become an important concern for global freshwater aquaculture (2, 3).

Species belonging to the genus *Enterococcus* are Gram-positive, facultatively anaerobic bacteria widely distributed in aquatic environments and in the gastrointestinal microbiota of animals (4, 5). Although many *Enterococcus* species are regarded as commensals, several members have emerged as opportunistic pathogens capable of infecting both terrestrial and aquatic hosts. In fish, *Enterococcus* infections have been associated with septicemia, hemorrhagic lesions, tissue necrosis, and significant mortality under stressful environmental conditions (6). Among them, *Enterococcus gallinarum* has attracted increasing attention owing to its zoonotic significance, intrinsic antimicrobial resistance, biofilm-forming ability, and capacity to persist under diverse environmental conditions (7, 8). *E. gallinarum* has traditionally been considered an opportunistic enterococcal species, but its detection in aquatic and human-associated environments warrants attention in the context of antimicrobial resistance. The occurrence of antimicrobial-resistant enterococci in aquaculture systems may have implications beyond fish health because aquatic environments can act as reservoirs and potential interfaces for the dissemination of antimicrobial-resistance determinants (9, 10). Therefore, characterization of antimicrobial resistance in *E. gallinarum* is relevant not only for understanding bacterial disease in cultured fish but also for informing broader One Health surveillance. Despite the increasing recognition of enterococci as opportunistic pathogens, the association of *E. gallinarum* with disease in cultured freshwater fish and the corresponding host responses remain poorly characterized.

Bighead carp (*Hypophthalmichthys nobilis*) is an economically valuable freshwater species cultured extensively throughout Asia because of its rapid growth, efficient utilization of natural food resources, and important contribution to freshwater aquaculture production (11, 12). In sewage-fed aquaculture systems such as the East Kolkata Wetlands, fish are frequently exposed to fluctuating water quality, elevated organic loads, and high microbial diversity, collectively increasing susceptibility to opportunistic bacterial infections (13, 14). Disease outbreaks occurring in these ecosystems are often multifactorial, involving interactions between environmental stressors and pathogenic microorganisms (15, 16). Therefore, understanding the etiology of disease outbreaks and the corresponding host immune responses is essential for developing effective disease surveillance and management strategies.

Successful bacterial pathogens employ multiple virulence mechanisms to colonize host tissues, evade immune defenses, and establish systemic infection (17, 18). Characteristics including biofilm formation, hemolytic activity, swimming motility, antimicrobial resistance, and tissue invasiveness contribute substantially to bacterial persistence and pathogenicity. Concurrently, infected fish activate innate and adaptive immune pathways involving pattern-recognition receptors, inflammatory mediators, complement activation, antigen presentation, and stress-response proteins (19). Although these immune pathways have been extensively investigated for several major fish bacterial pathogens, little information is available regarding host immune modulation following infection with *E. gallinarum*. To further characterize the host response associated with *E. gallinarum* infection, we selected a panel of immune- and stress-related genes representing complementary components of the fish immune response. Heat shock protein 70 (Hsp70) was included as a cellular stress-response marker because infection and tissue injury can induce molecular stress responses. Complement component 3 (C3) was selected as a central component of the complement system and an important effector of innate immune defense. Toll-like receptor 4 (TLR4) and NOD1 were examined as representative pattern-recognition receptors involved in the detection of pathogen-associated molecular patterns and initiation of innate immune signaling. Nuclear factor kappa B (NF-κB) was included as a key transcriptional regulator associated with inflammatory and innate immune responses. Immunoglobulin M (IgM) was selected as a major systemic antibody associated with humoral immune defense, whereas major histocompatibility complex class II (MHC II) was included as an indicator of antigen presentation and adaptive immune activation. The chemokine receptor (CR) was examined to provide an indication of chemokine-mediated immune-cell recruitment and inflammatory signaling. These genes were selected to provide a targeted overview of cellular stress, pathogen recognition, inflammatory signaling, complement activity, antigen presentation, and humoral immune responses during *E. gallinarum*-associated infection in *H. nobilis* (20, 21). Comprehensive characterization of emerging bacterial pathogens requires the integration of microbiological identification, molecular phylogenetics, antimicrobial susceptibility profiling, virulence phenotyping, experimental pathogenicity, histopathology, and host molecular immune analyses (22, 23). Such multidisciplinary investigations not only establish the pathogen’s etiological role but also provide mechanistic insights into disease progression and host-pathogen interactions (24, 25). Furthermore, characterization of antimicrobial resistance profiles is increasingly important because environmental bacteria inhabiting aquaculture systems may serve as reservoirs of resistance determinants that compromise therapeutic efficacy (26, 27).

In the present study, we investigated a mass mortality event affecting bighead carp (*Hypophthalmichthys nobilis*) cultured in a sewage-fed aquaculture system of the East Kolkata Wetlands, India. The study aimed to address several specific research objectives: identify the main isolate linked to mortality through biochemical tests and molecular analysis; examine its phenotypic traits; and evaluate its pathogenic potential under controlled conditions, including histopathological changes and transcriptional responses in *H. nobilis*. To address this, the causative bacterial isolate was characterized using biochemical assays, 16S rRNA gene sequencing, and phylogenetic analysis. We determined its antimicrobial susceptibility and multiple antibiotic resistance index, along with *in vitro* phenotypic characteristics including biofilm formation, swimming motility, and hemolytic activity. Experimental infection trials were subsequently conducted to assess pathogenic potential, and histopathological examinations were performed to characterize infection-associated tissue alterations. In parallel, we quantified the expression patterns of selected immune-related genes by quantitative real-time PCR in the brain, liver, kidney, and muscle. Rather than seeking to establish a novel molecular mechanism of virulence or immune evasion, the study integrates field observations, bacterial characterization, experimental infection, tissue pathology, and targeted immune-gene expression to provide a disease-associated characterization of *E. gallinarum* in bighead carp. The findings provide a basis for improving the diagnosis, surveillance, and health management of bacterial diseases in freshwater aquaculture.

## Materials and methods

### Collection of diseased fish

Between March and April 2023, a field investigation was conducted to examine recurrent mortality events in bighead carp (*Hypophthalmichthys nobilis*) (standard length: 40.2 ± 2.9 cm; body weight: 850.42 ± 3.8 g), cultured in Sardar bheri located in the East Kolkata Wetland (Lat: 22°56′ N and Long: 88°44′ E), West Bengal, India. This region comprises a mosaic of aquaculture ponds and sewage-fed wetlands that support extensive and semi-intensive fish farming. These culture systems primarily receive water through sewage inflows and interconnected local canal networks. Farmers operating these systems reported repeated episodes of fish mortality, prompting a detailed investigation. One sewage-fed wetland culture system that had recently experienced unexplained mortality was selected for intensive study. The mortality of *H. nobilis* was reported to be particularly severe, with passive surveillance data indicating an estimated cumulative mortality of approximately 50%. In response, researchers from the ICAR-Central Inland Fisheries Research Institute (ICAR-CIFRI), Barrackpore, India, the national nodal agency for fish disease surveillance, visited the affected site to assess the outbreak. Clinical examination of diseased fish revealed lethargy, anorexia, deep ulcerative lesions, and hemorrhages on both the lateral and ventral body surfaces. These manifestations suggested possible involvement of environmental stressors and/or infectious pathogens. A total of 20 symptomatic moribund *H. nobilis* exhibiting characteristic clinical signs were collected from the affected culture system. In parallel, 20 apparently healthy fish lacking visible clinical symptoms were sampled from separate culture facilities and served as asymptomatic controls. Concurrently, physicochemical parameters of pond water and sediment were assessed to evaluate environmental conditions associated with the mortality event. Following collection, fish were transported live to the laboratory for detailed pathological and microbiological investigations. Necropsies were performed, and fresh skin and gill smears were examined microscopically to detect the presence of ectoparasites. Owing to the suspicion of microbial infection, further bacteriological analyses were undertaken. The fish were then euthanized by an overdose of MS222 (150 mg/L for ~2 min) (Sigma-Aldrich), and post-mortem exams were carried out to document various clinical signs in the internal organs. Tissue samples from the brain, liver, kidney, and muscle of both symptomatic and asymptomatic fish were preserved in RNAlater for molecular analyses. In addition, liver, kidney, muscle, and spleen tissues were fixed in 10% neutral-buffered formalin for histopathological evaluation. The field investigation was designed to identify bacterial and other pathological conditions associated with the mortality event; therefore, the isolation of *E. gallinarum* was interpreted in conjunction with the clinical, histopathological, environmental and experimental infection findings rather than as evidence of a single-agent field outbreak. *E. gallinarum* was recovered from 15/20 (75%) symptomatic fish but was not recovered from any of the 20 apparently healthy fish examined (0/20). The animal use protocol was approved by the Institutional Animal Ethics Committee (IAEC) of ICAR-CIFRI, Kolkata, India (CIFRI-IAEC/17/2023-24. The study is documented in accordance with ARRIVE guidelines.

### Assessment of environmental parameters

In order to analyze the impact of environmental stress on the physicochemical characteristics of aquatic ecosystems, sediment and water samples were systematically collected from nine designated sites within the wetland. At each site, three sediment samples and three water samples were collected, for a total of 27 samples. These samples were then pooled (3 samples/site), resulting in a final set of 9 sediment and 9 water samples (25). The water temperature, pH, dissolved oxygen (DO), specific conductivity, total dissolved solids (TDS), and salinity were analysed with a probe (AQUAPROBE AP 7000, AlphaOmega Spain). We conducted an analysis of various water quality parameters, including total hardness, total alkalinity, chloride, biochemical oxygen demand (BOD), sulphate, total nitrogen, NO_3_^-^ -N (nitrate), NH_4_^+^ -N (ammonia), available phosphorous, total phosphorous, silicate (Si), calcium (Ca), magnesium (Mg), and total chlorophyll, using volumetric methods based on the standard procedures outlined in APHA 2017. Chlorophyll a was estimated using the non-acidification spectrophotometric method described by Jeffrey and Humphrey (28).

For the sediment quality assessment, we started by drying the sediment samples naturally in the shade at room temperature. Then, we gently ground them using a wooden hammer and carefully sieved them through a No. 10 (2 mm) mesh to ensure consistency. The samples were then stored safely in plastic packets. For measuring pH and conductivity (EC), we mixed the sediment with distilled water at a ratio of 1:2.5 and used a pH meter to check the pH levels. To determine the amount of sediment-available phosphorus (P), we employed the Molybdate blue method with a spectrophotometer, ensuring accurate results. The permanganate method was used to estimate available nitrogen (N) in wetland sediments (29). The Walkley and Black (30) method was used to analyze organic carbon (OC) in the sediment samples.

### Bacterial isolation

The method described by Kumar et al. (31), with minor modifications, was used to determine the cultivable bacterial load and recover bacterial isolates from the sampled fish tissues. Following external disinfection with 70% ethanol, individual fish were dissected aseptically (32). Muscle and fin tissues were collected and processed as described below. The sampled tissues were pooled (pooling was performed within each individual fish), chopped into small pieces and homogenized aseptically in 8 mL of sterile distilled water for 20–30 s at room temperature. The homogenate was serially diluted using sterile 1× PBS, and 100 µL of each dilution was spread onto Tryptic Soy Agar (TSA). Plates were incubated at 28 °C for 18–20 h. Plates containing 30–300 colonies were used for enumeration of cultivable bacteria. Representative colonies showing distinct morphological characteristics were selected for purification and subsequent biochemical and molecular identification. The isolation results were interpreted in relation to the clinical status of the fish.

### Molecular identification and phylogenetic analysis

Genomic DNA extraction from an isolated bacterium was performed as described by Sambrook and Russell (33). The DNA sample with a 260/280 ratio was chosen for further analysis. The isolated DNA was PCR (Thermal cyclers 9700, Applied Biosystems, USA) amplified by using 16S rRNA (e.g., forward and reverse primer sets) gene (34) (**Table 1****).** The PCR master mixture was prepared as described by Kumar et al. (31). Herewith, PCR products were run on a 1.8% agarose gel and visualised using the Gel Doc system (BIO-RAD, USA). The amplified PCR products were sequenced in both the forward and reverse directions using an ABI 373xl capillary sequencer (Applied Biosystems, USA). Using DNA Baser 7.0.0, the forward and reverse fragmentary sequences were aligned to create the contig. The 16S rRNA sequences of the assembled bacterium were aligned and compared with available NCBI GenBank sequences using BLAST (http://blast.ncbi.nlm.nih.gov). Afterwards, the sequence was submitted to GenBank to construct a phylogenetic tree.

**Table 1:** Primer sequences and qPCR amplification characteristics of target and reference genes used for RT-qPCR analysis in *H. nobilis*.

Target gene	Sequence (5′−3′)	Annealing temperature	References
*16S rRNA*	F-GTTGATCATGGCTCAG R-GGTTCACTTGTTACGACTT	52 ^0^C	(35)
*β -Actin*	F: GAC TTC GAG CAG GAG ATG G R: CAA GAA GGA TGG CTG GAA CA	55 °C	(36)
*Ef-1α*	F: AGGTGATTATCCTGAACCATCC R: AAAGGTGGATAGTCTGAGAAGC	58 °C	(37)
*C3*	F: TTGGCTGGACTGTGAAACCA R: AGGTGTATGATCCCACTTCCC	55 °C	(38)
*Hsp70*	F: CATGTGAGCGAGCCAAGAGA R: TTCAAAGCGAGCTCTGGTGA	60 °C	(39)
*TLR4*	F: ATGATGGAGCGCAATGCCAA R: ATGTTACTCAAAGGGTCTCTGCTCC	55 °C	(40)
*IgM*	F: TCATGATGATAAAGATGTAATGCGT R: TAATTTCCCGCCTTGTGCTC	55 °C	(41)
*NOD1*	F: GTTGGTGGGAAATACCTTGCC R: TGCTTTCGCCAGACTTCTTCC	56 °C	(40)
*MHC II*	TGGCCCTGACTGAACCACTG TCAGACCCACGCCACAGAAC	56 °C	(42)
*CR*	ACAGAGCCGATCTTGGGTTACTTG TGAAGGAGAGGCGGTGGATGTTAT	58 °C	(43)
*NF-kb*	AGGAGCAAAGATTAACATGCCAGA GCAAAGTGAAGAGGACTGTTGC	57 °C	

The sequences were aligned in MEGA11 (44) and using the Neighbor-Joining method (NJ), the evolutionary history of a recovered bacterium was created with the optimal tree (45). The tree is drawn to scale, with the length of the branch in the same units as the evolutionary distances used to conclude the phylogenetic tree. The maximum Composite Likelihood method was used to calculate the evolutionary distances (46). The analysis was based on 38 nucleotide sequences, and units represent the number of base substitutions per site. The codon positions were 1^st^ + 2^nd^ + 3^rd^ + noncoding, with ambiguous positions removed for each pair of sequences using the pairwise deletion option, resulting in a total of 1525 positions in the final dataset and Interactive Tree of Life (iTOL v4) were used for phylogenetic tree preparation (47).

### Biochemical assay

At first, the isolated strain was identified as either Gram-negative or Gram-positive by Gram staining. Later, the bacterial isolate were subjected to biochemical characterization following standard procedure, viz., ONPG (ββ-galactosidase), lysine utilization, ornithine utilization, urease, phenylalanine utilization, nitrate reduction, H_2_S production, citrate utilization, Voges Proskauer’s (VP), methyl red, indole, malonate utilization, esculin hydrolysis, arabinose, xylose, adonitol, rhamnose, cellobiose, melibiose, saccharose, raffinose, trehalose, glucose, lactose, and oxidase (KB003, Hi-media). The experiment was evaluated under sterile conditions and conducted five times independently.

### Antibiotic susceptibility test

The antimicrobial susceptibility profile of the isolated *Enterococcus gallinarum* strain was determined using the agar disk diffusion method (48, 49). The isolate was cultured in Mueller-Hinton broth, and the bacterial suspension was prepared for inoculation of Mueller–Hinton agar plates. We used commercial antibiotic disks (HiMedia, India) containing the specified concentrations for susceptibility testing. The plates were incubated at 37 °C for 24 h, following which the diameter of the inhibition zone surrounding each antimicrobial disk was measured in millimetres.

Susceptibility categories were assigned as susceptible (S), intermediate (I), or resistant (R) only when an appropriate, validated interpretive breakpoint for *Enterococcus* spp. was available. For antimicrobial agents for which an applicable enterococcal breakpoint could not be established, the observed inhibition-zone diameter was reported without assigning an S/I/R category and was designated as not interpreted (NI) (50, 51). We considered the applicability of the interpretive criteria to *E. gallinarum* and the isolate’s aquatic origin when revising the susceptibility classifications. Twenty-six antimicrobial agents were examined: ampicillin (25 µg), tetracycline (10 µg), erythromycin (10 µg), dicloxacillin (1 µg), streptomycin (25 µg), doxycycline (10 µg), ofloxacin (2 µg), amoxycillin (30 µg), ceftazidime (30 µg), cefixime (5 µg), rifampicin (5 µg), nalidixic acid (30 µg), piperacillin (10 µg), chloramphenicol (30 µg), polymyxin B (300 µg), colistin (10 µg), imipenem (10 µg), trimethoprim (5 µg), ciprofloxacin (5 µg), netilmicin sulphate (30 µg), tobramycin (10 µg), cefepime (30 µg), gentamicin (10 µg), fosfomycin (200 µg), nitrofurantoin (200 µg) and vancomycin (30µg), together with the antimicrobial panel used in the original assay. The disk potencies used in the experiment were recorded as specified by the manufacturer. The multiple antibiotic resistance (MAR) index of the isolate was estimated according to Krumperman (52). The experiment was assessed in sterile conditions with five biological replicates.

According to Krumperman (52), the Multiple Antibiotic Resistance (MAR) index is calculated as:


$$MAR\;Index=\frac{\text{a}}{\text{b}}$$

Where *a* represents the number of antibiotics to which the bacterial isolate showed resistance, and *b* represents the total number of antibiotics tested. A MAR index greater than 0.2 generally indicates bacterial isolates from environments with frequent or high antibiotic exposure.

### Survival assay

The care and treatment of experimental animals were conducted in accordance with recommendations set out by the Organisation for Economic Cooperation and Development (OECD). The Institutional Animal Ethics Committee, ICAR-Central Inland Fisheries Research Institute, Kolkata, India, authorised the animal utilisation procedure (CIFRI-IAEC/17/2023–24 for use in the experimental setting.

Healthy 250 numbers of *Hypophthalmichthys nobilis* fingerlings (average length: 15.3 ± 2.6 cm and average weight: 35.34 ± 3.06 gm) were procured from a local fish farm. The experiment was conducted on fish that seemed to be healthy and normal, displaying no apparent signs of illness such as ulcerations, hemorrhagic lesions, discoloration, loss of scales and redness on the outer body parts. In brief, the commonly encountered bacterial pathogens, such as *Flavobacterium* sp., *Aeromonas* sp., *Edwardsiella* sp., and *Pseudomonas* sp., were detected in the carps using PCR screening with primer sets specific to virulence genes (53, 54). Prior to the experimental setup, the carps were acclimatized in 200 L Fibre-reinforced plastic (FRP) tanks for 14 days. During this period, the carps were fed twice daily with commercial feed and maintained a constant photoperiod of 12 h: 12 h (light: dark). Later, *H. nobilis* fingerlings were transferred to glass aquaria containing 20 fish per tank. The bacterial strain was cultured in 20 mL sterile Tryptone soya broth (TSB) in a 50 mL Erlenmeyer flask (Himedia, India) for 24 h at 28 °C. The bacterial cell pellet was collected by centrifugation for 5 min at 5000 rpm and washed three times with sterile normal saline solution. Afterwards, the pellets were resuspended in saline solution, and the number of cells (CFU/ml) was estimated through the spread plate method. The experimental fish (100 fish, 20 fish/tank) were then injected intraperitoneally with 200 µl of the bacterial suspension (10^7^ colony-forming units/mL). Control fish (100 fish, 20 fish/tank) received an equivalent volume (200 µL) of sterile saline. Both the injected and non-injected carps were then placed in glass aquaria and observed every 24 h until 168 h. The fish were monitored at regular intervals for behavioural and external clinical signs, including reduced activity, abnormal swimming, loss of appetite, surface lesions, hemorrhage, and progressive morbidity (**Figure 1**). To confirm Koch’s postulate, the bacteria were reisolated and identified from the liver, kidney and blood of the moribund fish. Each experimental treatment was conducted with five independent biological replicates.

**Figure 1:**
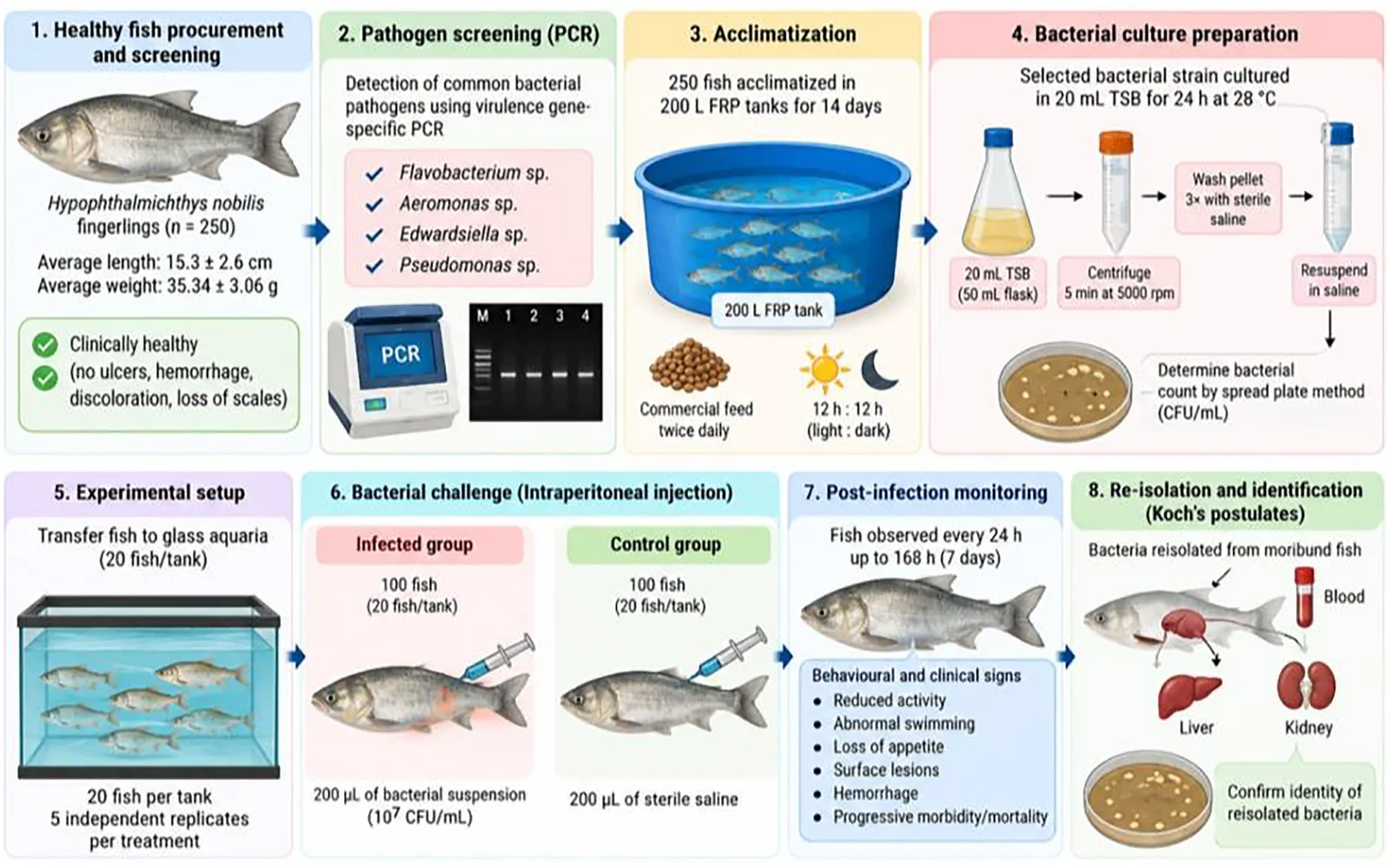
Experimental infection and pathogenicity assessment of isolated bacterial pathogen *Enterococcus gallinarum* against *Hypophthalmichthys nobilis*.

### Histopathology

For histopathological assessment, liver, kidney, skeletal muscle and spleen tissues were collected from [five] symptomatic/infected and [five] asymptomatic/non-infected fish. Tissues were fixed in 10% neutral-buffered formalin for 24 h, processed using standard dehydration, clearing and paraffin-embedding procedures, sectioned at 5 μm thickness, and stained with hematoxylin and eosin (H&E), as previously described (55, 56). The stained sections were examined using a light microscope (Leica EG 1140H, Germany). For quantitative assessment, non-overlapping fields from sections per fish were examined at 10 to 40 x magnification. Histological evaluation was performed by an observer blinded to the identity of the experimental group. Lesions were assessed using a predefined semi-quantitative scoring system: 0 = absent, 1 = mild, 2 = moderate, and 3 = severe.

For the liver, hepatocellular degeneration/vacuolation, sinusoidal congestion and necrosis were scored. For the kidney, tubular degeneration/necrosis and inflammatory-cell infiltration were evaluated. For skeletal muscle, we assessed myofibrillar degeneration/fragmentation and separation or disruption of muscle fibres. For the spleen, lymphoid depletion, melanomacrophage aggregation, vascular congestion and inflammatory infiltration were scored. For each fish, we calculated the mean lesion score from the examined microscopic fields and used it as the statistical unit. Lesion prevalence was expressed as the proportion of fish exhibiting a particular lesion. We compared lesion scores between symptomatic/infected and asymptomatic/non-infected fish, with *P* < 0.05 considered statistically significant.

For each fish, calculate an organ-specific mean score:


$$\text{Organ lesion scores}=\frac{\sum \text{ individual leasions scores}}{\text{number of lesions assessed}}$$

The symbol ∑ is called Sigma; it means sum or add all the values together.

### RNA isolation and cDNA synthesis

The total RNA was isolated using TRIzol^®^ reagent according to the manufacturer’s standard protocol. Briefly, brain, liver, kidney, and muscle tissues were collected from nine asymptomatic and symptomatic fish samples, pooled with three fish/replicate in three replicates, and immediately frozen in liquid nitrogen and stored at -80 °C (13, 2025). Total RNA was extracted from 100 mg of tissue using TRIzol^®^ reagent (Sigma, USA) according to the manufacturer’s protocol, with minor modifications. Briefly, tissues were homogenized in 1 mL chilled TRIzol^®^ and incubated at room temperature for 5 min. Chloroform (300 µL) was added, and the mixture was vigorously mixed, incubated for 10 min, and centrifuged at 12, 000 rpm for 20 min at 4 °C. The aqueous phase was transferred to a fresh tube and mixed with 700 µL isopropanol to precipitate RNA. Following incubation at -20 °C for 2 h, samples were centrifuged at 12, 000 rpm for 20 min at 4 °C. The resulting RNA pellet was washed twice with 80% ethanol, centrifuged at 10, 000 rpm for 10 min, air-dried, and resuspended in 50 µL DEPC-treated nuclease-free water. RNA samples were treated with RNase-free DNase I (Thermo Scientific, India) to eliminate residual genomic DNA contamination and stored at -20 °C until further use. RNA concentration and purity were determined using a NanoDrop spectrophotometer (Thermo Scientific, India) by measuring absorbance at 260 and 280 nm, while RNA integrity was assessed by electrophoresis on a 2% agarose gel.

First-strand complementary DNA (cDNA) was synthesized from total RNA using the RevertAid™ H Minus First Strand cDNA Synthesis Kit (Thermo Scientific, India; Cat. No. K1631) following the manufacturer’s protocol. Briefly, 1 µg of DNase-treated total RNA was mixed with 1 µL of random hexamer primers and nuclease-free water to a final volume of 10 µL. The reverse transcription reaction was prepared by adding a reaction mixture containing 20 U RiboLock RNase inhibitor, 2 µL of 10 mM dNTP mix, 200 U RevertAid™ H Minus M-MuLV reverse transcriptase, and 4 µL of 5× reaction buffer. The reaction mixture was gently mixed, briefly centrifuged, and incubated under the recommended thermal conditions to facilitate first-strand cDNA synthesis. Following reverse transcription, the enzyme was inactivated by heating the reaction at 70 °C for 5 min. The synthesized cDNA was immediately cooled on ice and subsequently stored at -20 °C until further use. The resulting cDNA served as the template for downstream PCR and quantitative gene expression analyses.

### Quantitative real-time PCR

The expression levels of heat shock protein 70 (*Hsp70*), complement component 3 (*C3*), chemokine receptor (*CR*), Toll-like receptor 4 (*TLR4*), nuclear factor kappa B (*NF-κB*), immunoglobulin M (*IgM*), major histocompatibility complex class II (*MHC II*), and nucleotide-binding oligomerization domain-containing protein 1 (*NOD1*), together with the reference genes *β-actin* and elongation factor 1-alpha (*EF-1α*), were quantified by RT-qPCR using a StepOnePlus Real-Time PCR System (Applied Biosystems) (N. 31) (**Table 1**). β-actin and EF-1α were used as reference genes for normalization. For each biological replicate, the relative expression quantity of each reference gene was calculated as 2^^Cq^, and the geometric mean of the two reference-gene quantities was used as the normalization factor:


NF=2^Cqβ−actin x 2^CqEF−1α 

The target-gene expression was normalized against this geometric mean, and relative expression between symptomatic and asymptomatic fish was calculated using the comparative Ct (2^^ΔΔCt^) method (57).

qPCR was performed in a total reaction volume of 20 µL containing 1 µL cDNA template (50 ng), 10 µL of 2× Maxima SYBR Green/ROX qPCR Master Mix (Thermo Fisher Scientific), 0.5 µL each of forward and reverse gene-specific primers, and nuclease-free water to the final volume. All reactions were carried out in triplicate for each biological replicate to ensure analytical reproducibility. Amplification of target immune-related genes and the reference (housekeeping) gene was conducted using the following thermal cycling conditions: an initial denaturation step at 95 °C for 10 min, followed by 40 cycles of denaturation at 95 °C for 15 s, annealing at 60 °C for 30 s, and extension at 72 °C for 30 s. All primer pairs generated a single distinct melting peak, with no detectable primer-dimer or nonspecific amplification. The reactions were subsequently cooled to 4 °C. For each primer pair, a no-template control (NTC) containing all reaction components except the cDNA template was included to monitor potential contamination and non-specific amplification. Additionally, a no-RT control was kept; it contains RNA that goes through the reaction without reverse transcriptase and is then subjected to qPCR (helps establish whether amplification arises from contaminating genomic DNA rather than cDNA).

The expression levels of the target genes were analyzed using the comparative CT method (2^^ΔΔCt^), following Livak and Schmittgen’s protocol (57). The Pfaffl relative standard curve method was employed to confirm the results (58, 59). The 2^^ΔΔCT^ values, log-transformed, were subjected to a t-test, and *P* values < 0.05 were considered statistically significant. For the field-based gene-expression analysis, tissues from nine symptomatic and nine asymptomatic fish were analysed. For each group, tissues from three individual fish were pooled to constitute one biological replicate, resulting in three independent biological replicates per group (n = 3). Each biological replicate was analysed in technical triplicate for each target and reference gene. The statistical unit was the biological replicate rather than the individual qPCR reaction.

### Biofilm assay

The biofilm assay was performed following the work of Kumar et al. (35) with slight modifications. Briefly, 250 µL aliquots of bacterial suspensions were transferred into the wells of a sterile 96-well plate (Catalogue No. 980040, Tarsons, India), and the OD_570_ value was adjusted to 0.5. The sterile TSB was used as a negative control. To form the biofilm, the plate was incubated for 24 hours at 28 °C. Afterwards, the non-adherent cells were washed twice with 300 µL of sterile 1X phosphate buffer saline (PBS, HiMedia, India). Absolute methanol (M1775, Sigma-Aldrich, USA) was added to the wells, which were then incubated at room temperature for up to 2 hours. Later, methanol from each well was discarded, and the wells were allowed to dry at room temperature (RT) overnight. The wells were stained with 0.1% crystal violet (200 µL) for 20 minutes at RT, washed with tap water under a moderate stream, and left to air-dry at RT. To remove the dye bound with adhering bacterial cells, 100 µL of 95% ethanol was added to each well. Finally, absorbance was measured at OD570 nm using a spectrophotometer. The experiment was performed in sterile conditions with five replicates.

### Swimming motility

The swimming motility in the bacterial isolate was analyzed as described by Tran et al. (60). In summary, the OD_550_ value of the bacterial suspension was initially set to 0.5, and 3 µL aliquots from the suspension were applied as a spot directly in the centre of freshly prepared 0.5% nutrient agar plates aseptically, followed by incubation for 24 h at 28 °C. Following incubation for the specified time period, the motility diameters contained within the plates were measured. The assay procedure was performed in triplicate alongside sterile nutrient agar as the corresponding control. The experiment was conducted under sterile conditions and independently, with five replicates.

### Hemolytic activity

The hemolytic activity of the bacterial isolate was assessed according to a standard protocol with slight modifications (61). Briefly, the Tryptone soya agar (TSA) plates were prepared by supplementing 5% defibrinated sheep blood. The pure stock cultures of *E. gallinarum* were grown overnight in TSB at 37 °C under constant agitation. Later, the overnight culture was diluted to an OD600 of 0.5, and 2 µl of the diluted culture was spotted in the middle of the haemolysin test plates. The plates were incubated at 37 °C for 48 h, after which the diameters of the clearing zones were measured. The assays were done with freshly prepared media in five replicates.

### Statistical analysis

Survival following experimental challenge was analysed using the Kaplan–Meier method, and survival distributions between treatment groups were compared using the log-rank (Mantel-Cox) test. Five independent replicate tanks were maintained for each treatment, with 20 fish per tank. The tank was considered the experimental unit for survival analysis to account for the non-independence of fish housed within the same tank. Fish that remained alive at the end of the 168-h observation period were treated as right-censored observations. Statistical analyses were performed using appropriate survival-analysis procedures, and differences were considered statistically significant at *P* < 0.05. Relative gene expression was calculated using the 2^^ΔΔCt^ method, with the geometric mean of housekeeping genes (*β-actin* and *Ef-1α*) as the reference. Expression levels were expressed as fold changes relative to the control group, which was assigned a value of 1.0. Prior to analysis, gene expression data were log-transformed to improve normality. Differences between the control and treatment groups were assessed using Student’s t-test, and values were considered significantly different at *P* < 0.05.

## Results and discussion

### Assessment of the culture system associated with a fish mass mortality event

The physicochemical characteristics of the outbreak pond and control pond are presented in **Tables 2** and **3**. Most of the basic physical parameters were comparable between the two ponds. The pH was 7.58 ± 1.16 in the outbreak pond and 8.1 ± 1.28 in the control pond, while EC was 1512 ± 6.88 and 1859 ± 8.54 µS/cm, respectively. Similarly, water temperature (28.0 ± 0.59 vs 28.5 ± 0.98 °C), water transparency (33 ± 1.48 vs 42 ± 6.28 cm), and water depth (84 ± 1.86 vs 110 ± 3.07 cm) were within the acceptable range. However, several important water-quality parameters showed marked differences or undesirable levels in the outbreak pond. Specific conductivity was 590 ± 2.57 µS/cm, exceeding the reference range of 100–500 µS/cm. Dissolved oxygen (DO) was only 3.69 ± 0.02 ppm, compared with 6 ± 1.42 ppm in the control pond. In contrast, TDS was significantly higher at 425 ± 3.58 ppm than in the control pond (127 ± 9.17 ppm). Chloride and BOD were also elevated in the outbreak pond, at 246 ± 5.88 ppm and 39 ± 2.88 ppm, respectively, compared with 168 ± 6.94 ppm and 15.29 ± 3.27 ppm in the control pond. The nutrient profile revealed pronounced enrichment in the outbreak pond. Available phosphorus was 0.332 ± 0.002 ppm, compared with 0.01 ppm in the control pond, whereas total phosphorus was 0.952 ± 0.04 ppm compared with 0.05 ppm. Similarly, ammonia-N (NH_4_^+^-N) reached 0.12 ± 0.001 ppm in the outbreak pond, compared with 0.002 ppm in the control pond. The chlorophyll a concentration was also substantially higher in the outbreak pond (46.02 ± 6.67 mg/m³) than in the control pond (22.5 mg/m³), indicating elevated phytoplankton biomass and nutrient enrichment. In contrast, nitrate-N, sulphate, silicate-S, calcium, and magnesium were within the optimum range.

**Table 2:** The mean ± SE values of water quality parameters recorded in cultured water bodies associated with the disease outbreak.

Parameters	Outbreak pond	Control pond	Remarks
Depth (cm)	84 ± 7.86	110 ± 8.07	Ok
Transp.(cm)	33 ± 1.58	42 ± 6.28	Ok
Temp Air (°C)	28.0 ± 0.59	28.5 ± 0.98	Ok
Water (°C)	28.0 ± 0.25	28.5 ± 0.86	Ok
pH	7.72 ± 1.08	6.5 ± 0.98	Ok
Sp.cond.(µS/cm)	590 ± 2.57	100–500	High
D.O. (ppm)	3.69 ± 0.02	6 ± 1.42	Low
FreeCO_2_ (ppm)	1.2 ± 0.08	1.6 ± 0.27	Ok
TDS (ppm)	425 ± 3.58	127 ± 2.17	High
Total Alkalinity (ppm)	184 ± 2.51	176 ± 2.38	Ok
Total Hardness (ppm)	167 ± 5.31	351 ± 7.75	Ok
Chloride (ppm)	246 ± 5.88	168 ± 6.94	High
Biochemical oxygen demand (BOD)	39 ± 2.88	15.29 ± 3.27	High
Sulphate (ppm)	0.91 ± 0.02	0.86 ± 0.02	Ok
NO_3_^-^ -N (nitrate) (ppm)	0.042 ± 0.001	0.085 ± 0.001	Ok
Av. Phosphorus (ppm)	0.332 ± 0.002	0.01 ± 0.001	Very High
Total Phosphorus (ppm)	0.952 ± 0.04	0.05 ± 0.001	Very High
Total Nitrogen (ppm)	0.14 ± 0.001	1.28 ± 0.02	Ok
Silicate-Si (ppm)	0.176 ± 0.02	0.5 ± 0.04	Ok
NH_4_^+^ -N (ammonia) (ppm)	0.12 ± 0.001	0.002 ± 0.001	Very High
Calcium (Ca) (ppm)	24.85 ± 1.68	35 ± 2.64	Ok
Magnesium (Mg) (ppm)	21.67 ± 2.58	28 ± 2.48	Ok
Chlorophyll a (mg/m^3^)	46.020 ± 6.67	22.5 ± 1.29	Very High

**Table 3:** The mean ± SE values of sediment quality parameters recorded in cultured water bodies associated with the disease outbreak.

Parameters	Outbreak pond	Control pond	Remarks
pH	7.58 ± 1.16	8.1 ± 1.28	Ok
EC (µs/cm)	1512 ± 6.88	1859 ± 8.54	Ok
Organic Carbon (%)	5.7 ± 0.59	6.38 ± 1.34	Ok
Sand (%)	69 ± 1.24	59.18 ± 2.69	Ok
Silt (%)	17 ± 2.25	21.54 ± 3.08	Ok
Clay (%)	14 ± 2.39	20.38 ± 2.73	Ok
Free CaCo_3%_	16 ± 2.28	5.98 ± 1.29	High
Av. P (mg/100g)	2.536 ± 0.08	6.24 ± 1.09	Ok
Total P (mg/100g)	45.046 ± 2.58	27.49 ± 4.12	High
Av. N (mg/100g)	38.64 ± 1.98	20–50	Ok
Total N (%)	0.796 ± 0.001	0.38 ± 0.02	High

The soil characteristics showed that the outbreak and control ponds had broadly comparable textural properties. In the outbreak pond, sand, silt and clay constituted 69 ± 1.24%, 17 ± 2.25%, and 14 ± 2.39%, respectively, compared with 59.18 ± 2.69%, 21.54 ± 3.08%, and 20.38 ± 2.73% in the control pond. In contrast, the outbreak pond showed marked enrichment of several soil chemical parameters. Free CaCO_3_ was 16 ± 2.28%, substantially higher than 5.98 ± 1.29% in the control pond. Total phosphorus was 45.046 ± 2.58 mg/100 g in the outbreak pond compared with 27.49 ± 4.12 mg/100 g in the control pond, while total nitrogen was 0.796 ± 0.001% compared with 0.38 ± 0.02%, respectively. In contrast, available phosphorus and available nitrogen remained within the acceptable range. Overall, the outbreak pond exhibited poor water-quality conditions characterized particularly by reduced DO, elevated BOD, conductivity, TDS, chloride, ammonia-N, phosphorus and chlorophyll a. The concurrent enrichment of soil phosphorus and nitrogen, together with elevated free CaCO_3_, suggests substantial nutrient accumulation in the pond environment (2, 62). These conditions may promote excessive primary productivity and organic matter decomposition, potentially contributing to oxygen depletion and environmental stress on cultured fish.

### Characterization of *E. gallinarum* isolated from *H. nobilis*

Moribund *Hypophthalmichthys nobilis* collected during the mortality event exhibited characteristic clinical signs of bacterial septicemia, including extensive cutaneous hemorrhages, ulcerative lesions, scale loss, and body discoloration (**Figure 2A**), indicating severe systemic infection. A dominant bacterial isolate recovered from diseased fish was subjected to phenotypic and molecular characterization. Biochemical analysis demonstrated that the isolate was positive for ribose, xylose, adonitol, galactose, arabitol, arginine dihydrolase, trehalose, and Voges-Proskauer reactions, whereas negative reactions were observed for erythritol, D- and L-arabinose, inositol, starch, dulcitol, melibiose, inulin, D-raffinose, glycogen, fucose, alkaline phosphatase, indole, urease, and motility (**Table 4**). The observed biochemical profile was consistent with the diagnostic characteristics of *Enterococcus gallinarum* (64, 65). Molecular identification based on 16S rRNA gene sequencing further confirmed the isolate as *E. gallinarum* (accession number OR999570). Phylogenetic analysis showed that the isolate clustered closely with previously reported *E. gallinarum* strains from aquatic and environmental sources, forming a well-supported lineage within the *Enterococcus* genus (**Figure 2B**). This phylogenetic placement was consistent with the taxonomic identification of the isolate as *E. gallinarum* and indicated its close evolutionary relatedness to the reference strains (66).

**Figure 2:**
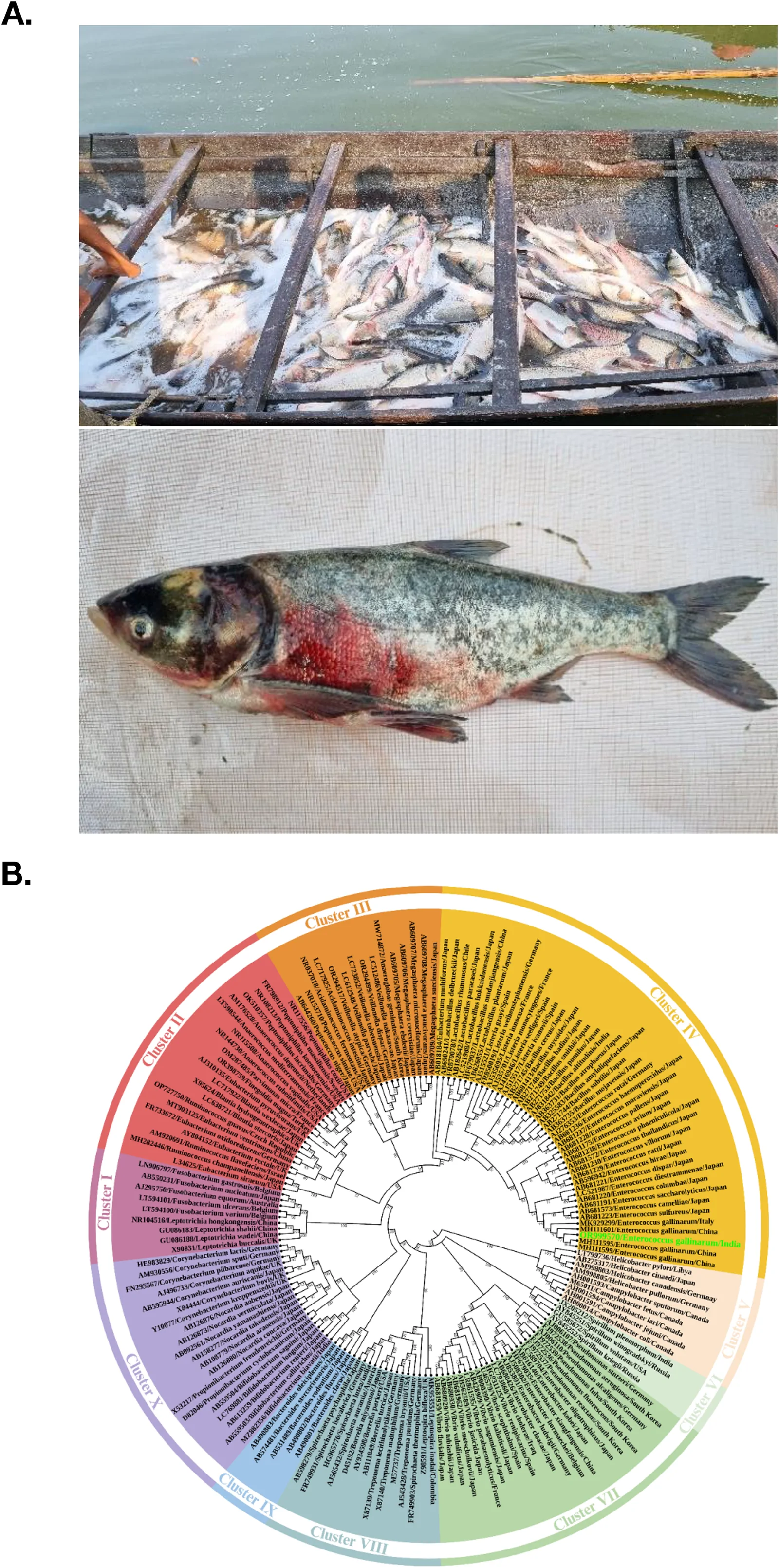
**(A)** Infected moribund *Hypophthalmichthys nobilis* samples showing clinical signs were collected from the culture facility. **(B)** Phylogenetic tree analysis of *Enterococcus gallinarum* based on 16S rRNA nucleotide sequences following the maximum composite likelihood method using MEGA11 software. The numbers next to the branches indicate percentages from 1000 bootstrap replicates. Bootstrap values above 50% are shown at the nodes. The isolate was categorised into 10 clusters, indicated by shades of different colours.

**Table 4:** Biochemical assay of *E. gallinarum* isolated from *H. nobilis.*.

Features	*E. gallinarum*
Erythritol	Negative^#^
D-arabinose	Negative^#^
L-arabinose	Negative^#^
Ribose	Positive^#^
Xylose	Positive^#^
Adonitol	Positive^#^
Galactose	Positive^#^
Inosotiol	Negative^#^
Starch	Negative^#^
Dulcitol	Negative^#^
Mellibiose	Negative^#^
Inulin	Negative^#^
D-raffinose	Negative^#^
Glycogen	Negative^#^
Arabitol	Positive^#^
Fucose	Negative^#^
Alkaline phosphatase	Negative^#^
Arginine dehydrolase	Positive^#^
Trehalose	Positive^#^
Indole	Negative^#^
Voges-Proskauer	Positive^#^
Urease	Negative^#^
Motility	Positive^#^

# The reference was subjected to the following standard protocol (63).

The isolate investigated in the present study was designated *E. gallinarum* IFEGHNEK1. Rather than considering isolation of this organism alone as definitive evidence of causation of the field outbreak, we evaluated its association with the mortality event using complementary evidence from experimental challenge, histopathology, and host-response analyses. This distinction is important because opportunistic bacteria may be recovered from diseased fish in association with environmental stressors or concurrent infections. Although members of the genus *Enterococcus* are widely recognized as commensal microorganisms inhabiting aquatic environments and the gastrointestinal tract of animals, several species have emerged as opportunistic pathogens capable of causing severe infections under stressful environmental conditions (67–69). The external clinical manifestations observed in infected bighead carp are typical of systemic bacterial septicemia and closely resemble pathological signs previously reported in enterococcal infections of freshwater fish. Such lesions are generally associated with bacterial invasion of host tissues, vascular damage, inflammatory cell infiltration, and progressive tissue necrosis, ultimately compromising fish health and survival (62, 70). The biochemical characteristics obtained in the present study agree well with previously described metabolic profiles of *E. gallinarum*. Positive utilization of multiple carbohydrates, including ribose, xylose, galactose, arabitol, and trehalose, indicates considerable metabolic versatility, enabling the bacterium to exploit diverse nutrient sources within both the aquatic environment and host tissues. Similarly, a positive Voges-Proskauer reaction indicates acetoin production *via* the butanediol fermentation pathway, a characteristic feature of many enterococci. Conversely, negative reactions for urease, indole production, starch hydrolysis, alkaline phosphatase, and motility effectively distinguished the isolate from several other Gram-positive fish pathogens, supporting accurate species identification (64, 65, 71). Molecular phylogenetic analysis further strengthened these findings by demonstrating close clustering with validated *E. gallinarum* reference strains, suggesting a high degree of genetic conservation despite geographical variation. A limitation of the investigation is that it did not include comprehensive metagenomic screening for all pathogens. While skin and gill smears were examined for ectoparasites and bacteriological tests were conducted, the presence of other infectious agents cannot be ruled out. The outbreak occurred in a sewage-fed system with environmental stressors likely contributing to disease susceptibility. The observed environmental disturbances, reduced dissolved oxygen and increased BOD, conductivity, TDS, chloride, ammonia-N, phosphorus, and chlorophyll a, may have acted as predisposing factors.

### Phenotypic properties of isolated bacterial pathogen

The antimicrobial susceptibility profile of the isolated *E. gallinarum* revealed a multidrug-resistant phenotype (**Table 5**, **Supplementary Table 1**). The isolate was susceptible to 17 of the 26 antibiotics tested, including ampicillin, cefixime, ceftazidime, ciprofloxacin, ofloxacin, gentamicin, colistin, kanamycin, streptomycin, cefepime, fosfomycin, and piperacillin. Intermediate susceptibility was observed for tetracycline and nitrofurantoin, whereas resistance was detected against doxycycline, erythromycin, dicloxacillin, polymyxin B, rifampicin, chloramphenicol, and imipenem. The isolate exhibited a MAR index of 0.27 (7 resistant/26 antibiotics), indicating that it originated from an environment exposed to considerable antibiotic selection pressure (**Table 6**). The susceptibility pattern was largely comparable to that of the reference *E. gallinarum* strain but showed additional resistance to doxycycline, polymyxin B, chloramphenicol, and imipenem, suggesting phenotypic adaptation of the field isolate. The elevated MAR index (>0.2) further indicates frequent exposure to antimicrobial agents, likely associated with the sewage-fed aquaculture environment (72, 73).

**Table 5:** Zone of inhibition diameter (mm) by the *E*. *gallinarum* isolated from infected fish.

Antibiotics	*E*. *gallinarum* (Isolated)	*E*. *gallinarum* (Standard)
Trimethoprim	S	S
Netilmicin sulphate	S	S
Ampicillin	S	S
Cefixime	S	S
Tetracycline	I	S
Doxycycline	R	S
Ceftazidime	S	S
Erythromycin	R	R
Ciprofloxacin	S	S
Ofloxacin	S	S
Dicloxacillin	R	R
Nalidixic acid	S	S
Gentamicin	S	S
Amoxycillin	S	R
Polymyxin B	R	S
Colistin	S	S
Kanamycin	S	S
Tobramycin	S	S
Streptomycin	S	S
Rifampicin	R	R
Nitrofurantoin	I	S
Piperacillin	S	S
Chloramphenicol	R	I
Cefepime	S	S
Fosfomycin	S	S
Imipenem	R	S

Following the guidelines of the Clinical and Laboratory Standards Institute (50, 51), susceptibility of recovered strains to different antibiotics is expressed as sensitive (S), intermediate (I) and resistant (R).

**Table 6:** MAR indices of *E*. *gallinarum* isolated from *H*. *nobilis*.

Isolated bacteria	MAR value
*E*. *gallinarum*	0.27

The resistance to several clinically important antibiotics is consistent with the growing occurrence of multidrug-resistant enterococci in aquatic ecosystems and highlights their potential role as reservoirs of antimicrobial resistance genes (74, 75). *Enterococcus* species possess remarkable genomic plasticity, enabling the acquisition of resistance determinants *via* plasmids, transposons, and other mobile genetic elements. Continuous exposure to antimicrobial residues from aquaculture practices, livestock waste, and sewage discharge exerts strong selective pressure, promoting the persistence and dissemination of resistant bacterial populations. The isolate’s resistance to clinically important antibiotics, including doxycycline, erythromycin, rifampicin, chloramphenicol, and imipenem, is therefore particularly concerning, as these antibiotics are widely used in both veterinary and human medicine. Despite the multidrug-resistant phenotype, the isolate remained susceptible to several β-lactams, aminoglycosides, fluoroquinolones, and cephalosporins. The observed susceptibility pattern provides a phenotypic description of the isolate but should not be interpreted as evidence of clinical or therapeutic efficacy, particularly because standardized enterococcal breakpoints are not available for all antimicrobial agents examined. The present study was based on disk diffusion testing and did not include MIC determination or experimental confirmation of individual resistance determinants.

The detection of antimicrobial resistance in *E. gallinarum* from a sewage-fed aquaculture system also has broader public-health and One Health implications. Sewage-fed aquaculture systems receive substantial inputs of microorganisms and microbial genetic material originating from domestic wastewater and other anthropogenic sources (76). Such environments may therefore provide opportunities for the persistence, exchange, and dissemination of antimicrobial-resistant bacteria and resistance determinants. The recovery of an *E. gallinarum* isolate resistant to 7 of the 26 antimicrobials tested, with a MAR index of 0.27, indicates that antimicrobial resistance is present within the bacterial population associated with this production system. Although the present study does not establish the origin or transmissibility of these resistance phenotypes, their occurrence in a wastewater-associated aquaculture environment warrants continued surveillance. The public-health relevance is particularly important because aquaculture systems can create interfaces among wastewater, aquatic animals, environmental microorganisms, and humans. Fish and farm workers may come into contact with contaminated water, sediment, or fish-associated bacteria, while resistant bacteria may potentially enter downstream environments through water discharge or movement of aquaculture products (77). However, the detection of resistant *E. gallinarum* in fish should not be interpreted as evidence of zoonotic transmission. The present study did not investigate transmission between fish and humans, identify resistance genes, or determine whether the observed resistance determinants are mobile or transferable. Similarly, the study does not establish whether the resistant isolate originated from sewage, the aquaculture environment, or the fish itself. Therefore, the antimicrobial susceptibility findings should be interpreted as an *in vitro* phenotypic profile rather than as a basis for selecting therapeutic agents. Nevertheless, the findings emphasise the need for routine antimicrobial susceptibility testing, judicious antibiotic use, and continuous surveillance to minimise the spread of multidrug-resistant *E. gallinarum* in freshwater aquaculture (78, 79).

### Virulence profile of isolated *E. gallinarum*

To determine whether the field-associated *E. gallinarum* isolate was capable of producing disease in *H. nobilis*, a controlled experimental challenge was conducted. Following exposure to *E. gallinarum*, mortality occurred rapidly, with all challenged fish dying by 24 h post-challenge, whereas no mortality was observed in the PBS-treated control group during the 168-h observation period. Survival distributions differed significantly between the two treatments (log-rank test, χ² = 9.00, P = 0.0027), with the tank considered the experimental unit (**Figure 3**). These findings demonstrate that the isolate was capable of causing mortality in *H. nobilis* under the experimental conditions used. Importantly, because the challenge involved an experimentally administered bacterial dose and an intraperitoneal exposure route, these results demonstrate pathogenic potential under controlled conditions but do not by themselves establish that *E. gallinarum* was the sole cause of the original field mortality event.

**Figure 3:**
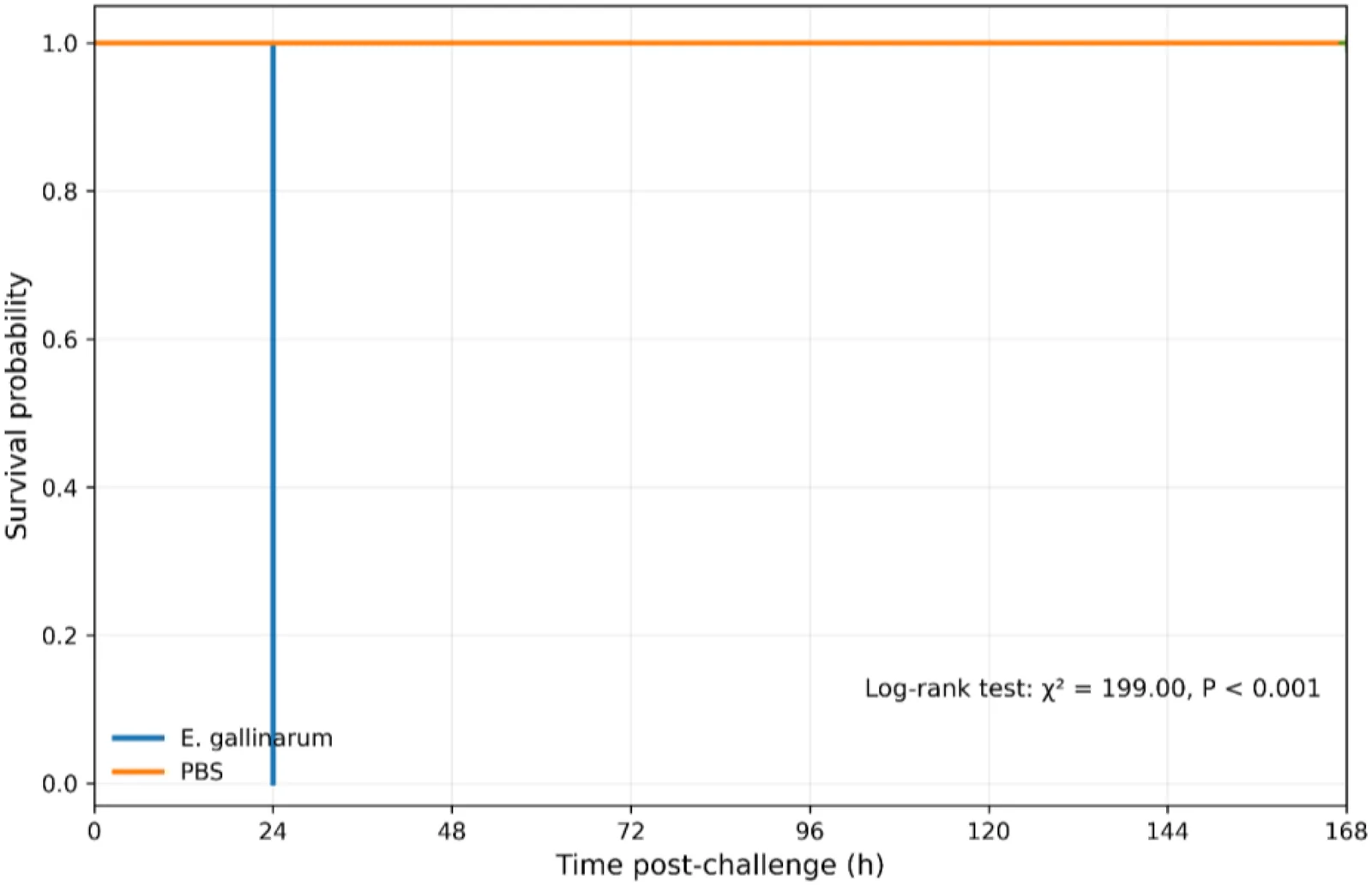
Kaplan-Meier survival curves of *Hypophthalmichthys nobilis* following experimental challenge with *Enterococcus gallinarum*. Survival was monitored for 168 h post-challenge in fish exposed to *E. gallinarum* and in the PBS control group, with five independent replicate tanks per treatment and 20 fish per tank. Complete mortality was observed in the *E. gallinarum*-challenged group by 24 h, whereas all fish in the PBS control group survived throughout the 168-h observation period. Differences in survival between treatments were assessed using the log-rank test, considering the tank as the experimental unit (χ² = 9.00, *P* = 0.0027).

Selected *in vitro* phenotypic characteristics potentially associated with virulence were evaluated in the isolated *Enterococcus gallinarum*, including biofilm formation, swimming motility and haemolytic activity (**Figure 4****;** **Table 7**). The isolate produced a crystal violet absorbance of 2.89 ± 0.48 at 570 nm in the biofilm assay, compared with 1.85 ± 0.07 for the *Aeromonas hydrophila* control, while the PBS control showed negligible staining. The isolate also exhibited a swimming migration diameter of 29 ± 2.9 mm, compared with 16 ± 3.3 mm for *A. hydrophila*. In the sheep-blood haemolysis assay, *E. gallinarum* produced a clear haemolytic zone of 25 ± 2.8 mm, with a haemolytic index of 4.2 ± 2.2. These findings demonstrate that the isolate possesses measurable *in vitro* biofilm-forming, motility and haemolytic phenotypes. However, differences between *E. gallinarum* and *A. hydrophila* in these assays should be interpreted cautiously because species-specific differences in growth characteristics, biomass production and physiological responses may influence the measured phenotypes.

**Figure 4:**
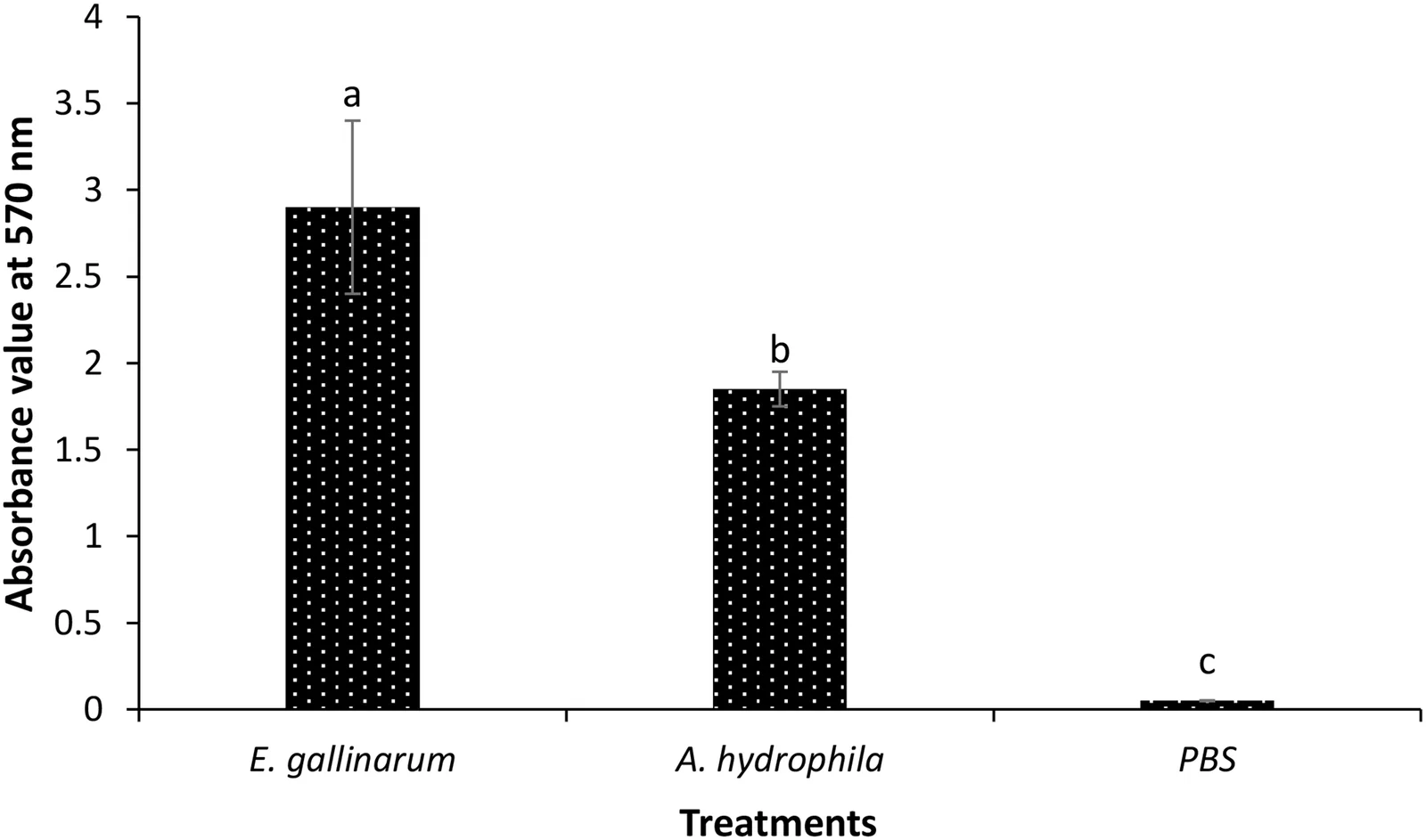
Biofilm formation assay on 96-well polystyrene plates for the isolated *E. gallinarum* bacterium. *Aeromonas hydrophila* was used as a positive control, while plates supplemented with PBS served as a negative control for the assay. Data are presented as the mean ± SE of five replicates and three independent experiments for biofilm formation. Different letters indicate significant differences (One-way ANOVA with Tukey’s *post hoc* test; *P* < 0.05).

**Table 7:** Swimming motility and haemolysin assay of *Enteroococcus gallinarum* isolated from infected *H*. *nobilis*.

Bacterial isolate	Swimming motility assay of bacterial strain (mm) (mean ± SE)	Hemolysin assay of bacterial strain (mm) (mean ± SE)		
		Clear zone diameter	Colony diameter	Ratio of clear zone and colony diameter
*E*. *gallinarum*	29 ± 2.9	25 ± 2.8	6 ± 3.4	4.2 ± 2.2
*A. hydrophila^#^*	16 ± 3.3	21 ± 2.4	5.5 ± 2.6	3.8 ± 2.8
PBS	0 ± 00	0 ± 00	0 ± 00	0 ± 00

The motility and haemolysin assay of bacteria is presented as the mean ± SE of six replicates and three independent experiments. *Aeromonas hydrophila* was used as a positive control, while plates supplemented with PBS served as a negative control for the assay.

The biofilm formation, swimming motility and haemolytic assays demonstrated distinct *in vitro* phenotypes of the recovered *E. gallinarum*. Biofilm formation may contribute to bacterial attachment and persistence under appropriate environmental conditions; however, the crystal-violet assay primarily measures surface-associated biomass and cannot, by itself, establish enhanced virulence or persistence within the fish host. Similarly, the greater swimming migration observed under the present assay conditions indicates a motility phenotype but does not directly demonstrate increased dissemination, tissue invasion or colonization *in vivo*. Comparisons with *A. hydrophila* should also be interpreted cautiously because differences in growth rate, biomass accumulation and species-specific physiology may influence these measurements (80, 81). The significantly greater biofilm-forming capacity observed in the present isolate compared with *A. hydrophila* suggests an enhanced ability to establish persistent infections in cultured fish. Similarly, the high swimming motility indicates an increased capacity for bacterial dissemination, facilitating tissue invasion and colonization during infection (82, 83).

The haemolytic activity observed on sheep blood agar indicates an *in vitro* haemolytic phenotype potentially associated with the production of erythrocyte-disrupting factors. However, haemolysis on sheep erythrocytes cannot be directly extrapolated to lysis of fish erythrocytes, tissue destruction or systemic invasion in *H. nobilis* (84, 85). Collectively, these findings demonstrate that the recovered *E. gallinarum* exhibits several *in vitro* phenotypes potentially associated with pathogenicity. These observations, together with the mortality observed following experimental challenge, provide complementary evidence of the isolate’s pathogenic potential; however, individual *in vitro* assays should not be interpreted as direct measures of virulence, immune evasion, systemic invasion, or tissue damage. Nevertheless, the findings highlight the emergence of *E. gallinarum* as an important opportunistic pathogen in freshwater aquaculture and emphasize the need for continuous monitoring of its virulence attributes to improve disease prevention and management strategies.

### Histopathological alterations during *H. nobilis* infection

Histopathological examination revealed marked tissue damage in the liver, kidney, muscle, and spleen of *Hypophthalmichthys nobilis* infected with *Enterococcus gallinarum* compared with the asymptomatic fish (**Figure 5**). Liver sections from healthy fish exhibited normal hepatic architecture with well-organized hepatocytes and intact sinusoids. In contrast, infected fish showed extensive hepatocellular degeneration, severe cytoplasmic vacuolation, sinusoidal congestion, and focal necrosis, indicating impaired liver function (**Figure 5A**). Similarly, kidney tissues of infected fish displayed pronounced degeneration of renal tubules, disruption of glomerular architecture, tubular necrosis, and inflammatory cell infiltration, whereas the kidneys of healthy fish maintained normal structural organization (**Figure 5B**). Skeletal muscle from infected fish exhibited extensive myofibrillar degeneration, fragmentation of muscle fibers, edema, and separation of muscle bundles, reflecting severe tissue destruction associated with bacterial invasion (**Figure 5C**). The spleen also showed marked pathological alterations, including melanomacrophage aggregation, vascular congestion, lymphoid depletion, and inflammatory infiltration compared with the normal splenic architecture observed in the non-infected group (**Figure 5D**).

**Figure 5:**
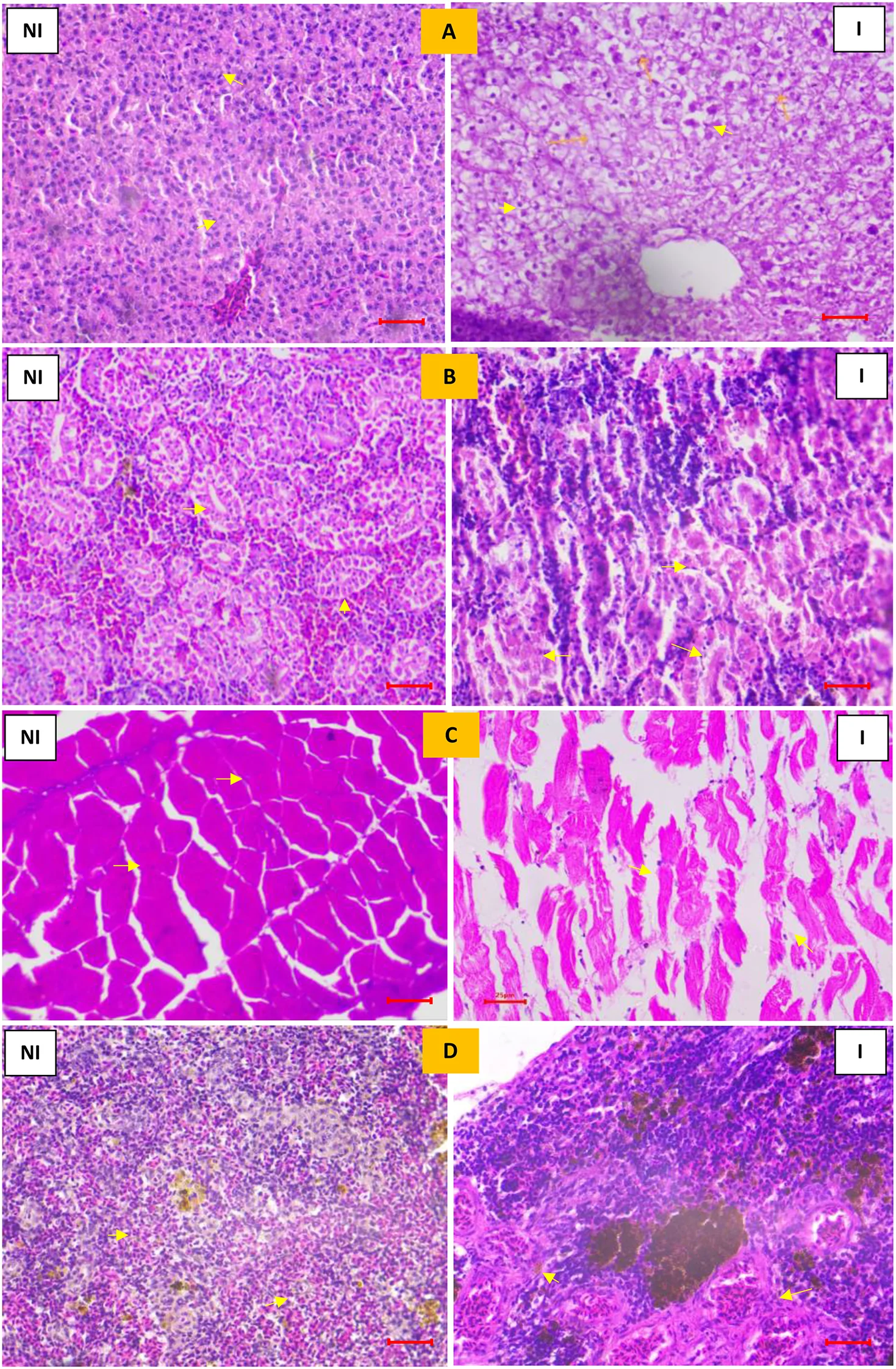
Quantitative and representative histopathological alterations associated with *Enterococcus gallinarum* infection in *Hypophthalmichthys nobilis*. Representative H&E-stained sections of **(A)** liver, **(B)** kidney, **(C)** skeletal muscle and **(D)** spleen from non-infected/asymptomatic (NI; left panels) and infected/symptomatic (I; right panels) fish. Yellow arrowheads indicate representative lesions: **(A)** hepatocellular degeneration/vacuolation, sinusoidal congestion and focal necrosis; **(B)** renal tubular degeneration/necrosis and inflammatory-cell infiltration; **(C)** myofibrillar degeneration, fragmentation and separation of muscle fibres; and **(D)** melanomacrophage aggregation, vascular congestion, lymphoid depletion and inflammatory infiltration. Scale bars = 25 μm. The arrowhead in the figures indicates modulation of *H. nobilis* tissue morphology during the disease outbreak.

Quantitative histopathological assessment demonstrated substantially greater tissue injury in the symptomatic/infected fish than in the asymptomatic/non-infected fish across the examined organs (**Table 8**). In the liver, infected fish showed higher scores for hepatocellular degeneration/vacuolation, sinusoidal congestion and necrosis than the non-infected fish (score 3 and *P* < 0.05). In the kidney, tubular degeneration/necrosis and inflammatory-cell infiltration were more frequently observed and had higher severity scores in infected fish (score 3 and *P* < 0.05). The skeletal muscle of infected fish exhibited increased myofibrillar degeneration and fragmentation, together with separation/disorganization of muscle fibres (score 2 and *P* < 0.05). In the spleen, infected fish showed increased lymphoid depletion, melanomacrophage aggregation, vascular congestion and inflammatory infiltration compared with non-infected fish (score 2 and *P* < 0.05). Overall, the quantitative lesion scores indicate that the infection-associated pathological changes involved multiple organs, consistent with a systemic pathological response.

**Table 8:** Semi-quantitative histopathological lesion-scoring system used for assessment of tissue damage in *Hypophthalmichthys nobilis* (0 = absent, 1 = mild, 2 = moderate, and 3 = severe).

Organ	Histopathological parameter	Assigned score (0–3)
Liver	Hepatocellular degeneration/vacuolation, Sinusoidal/vascular congestion, Hepatocellular necrosis	Score 3
Kidney	Renal tubular degeneration, Tubular necrosis, Inflammatory-cell infiltration	Score 3
Muscle	Myofibrillar degeneration, Myofibrillar fragmentation, Fibre separation/edema	Score 2
Spleen	Lymphoid depletion, Melanomacrophage aggregation, Vascular congestion, Inflammatory-cell infiltration	Score 2

The extensive histopathological lesions demonstrate that *E. gallinarum* induces systemic tissue injury following infection. The liver and kidney are primary target organs involved in metabolism, detoxification, and immune regulation; therefore, degeneration and necrosis of these tissues indicate severe impairment of physiological homeostasis (86, 87). Likewise, the disruption of skeletal muscle fibers is consistent with the external ulcerative lesions observed in diseased fish and suggests bacterial dissemination into peripheral tissues (88). The pronounced alterations in the spleen, a major lymphoid organ in teleosts, further indicate activation of systemic immune responses during infection (89). Such pathological changes are characteristic of septicemic bacterial infections and likely result from the combined effects of bacterial proliferation, hemolytic activity, inflammatory responses, and host tissue damage (90). Collectively, the quantitative histopathological findings demonstrate substantial multi-organ tissue injury associated with *E. gallinarum* infection under the experimental conditions. The involvement of hepatic, renal, muscular and splenic tissues is consistent with a systemic pathological response. However, histopathology alone cannot demonstrate bacterial localization within the lesions or establish direct tissue invasion. The present interpretation is therefore based on the combined evidence from experimental mortality, recovery of *E. gallinarum* from internal tissues, clinical manifestations and histopathological alterations.

### Impact of *H. nobilis* infection on host immune response

To elucidate the host immune response during *Enterococcus gallinarum* infection, the relative expression of immune- and stress-related genes, including *Hsp70, C3, CR, TLR4, NF-κB, IgM, MHC II*, and *NOD1*, was evaluated in the brain, liver, kidney, and muscle tissues of *Hypophthalmichthys nobilis* (**Figure 6**). Infection-induced tissue-specific modulation of innate and adaptive immune genes reflects a complex host response to bacterial invasion (91, 92). In the brain, infected fish showed significant upregulation of *C3, NF-κB*, and *IgM*, indicating activation of the complement cascade, inflammatory signaling, and humoral immunity. In contrast, the expression of *Hsp70, CR, TLR4*, and *MHC II* was markedly downregulated, while *NOD1* remained largely unchanged (**Figure 6A**). In the liver, a pronounced increase in *Hsp70*, *C3, NF-κB*, and *NOD1* expression was observed, whereas *CR, TLR4, IgM*, and *MHC II* were significantly suppressed (**Figure 6B**). Similarly, the kidney exhibited elevated expression of *Hsp70, NF-κB*, and *IgM*, accompanied by significant reductions in *C3, CR, TLR4, MHC II*, and *NOD1* (**Figure 6C**). The muscle tissue displayed the strongest immune activation, with substantial upregulation of *Hsp70, C3, CR, TLR4, NF-κB, IgM*, and *NOD1*, while *MHC II* expression was significantly downregulated (**Figure 6D**).

**Figure 6:**
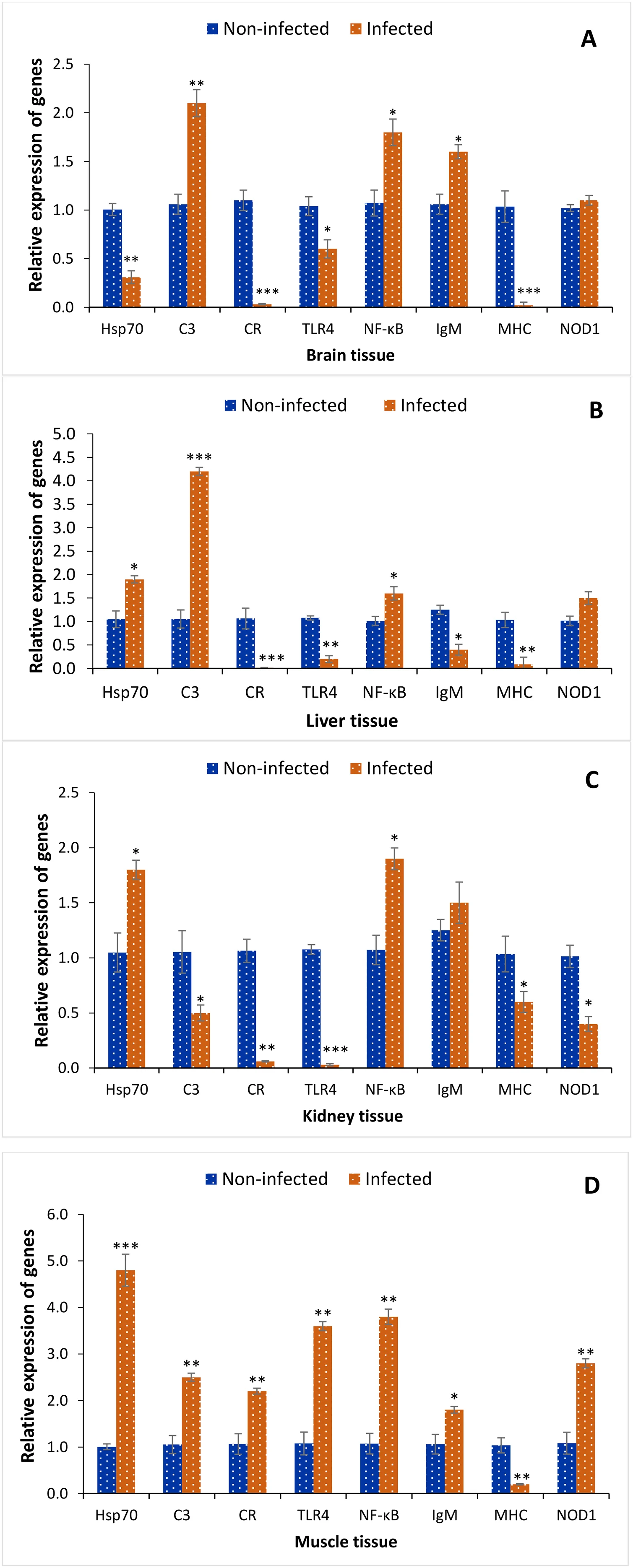
Fold change in gene expression of bighead carp *(Hypophthalmichthys nobilis)* tissue samples in non-infected and infected groups. Expression of heat shock protein 70 (*Hsp70*), complement system (*C3*), Chemokine receptors (*CR*), Toll-like receptors (*TLR4*), nuclear factor NF-κB (*NF- κB*), immunoglobulin M (*IgM*), major histocompatibility complex (*MHC II*) and NOD-like receptors (*NOD 1*). The temporal expression was analyzed from non-infected and infected bighead carp from **(A)** brain, **(B)** liver, **(C)** kidney, and **(D)** muscle tissue samples. The expression level in the non-infected group was regarded as 1.0, and thereby the expression ratio of the infected group was expressed in relation to the control group. The results are the mean ± SE (n=3), and the vertical bars with asterisks indicate significant differences between treatment groups (*P < 0.05, **P < 0.01, ***P < 0.001, ****P < 0.0001, *****P < 0.00001) between the non-infected and infected groups.

The differential expression of these immune-associated genes indicates a pronounced and tissue-specific transcriptional response in symptomatic *H. nobilis* during the mortality event (21). The increased expression of *Hsp70* was consistent with cellular stress, whereas elevated *C3* and *NF-κB* transcripts were associated with activation of complement- and inflammation-related responses (93, 94). Increased *IgM* expression may reflect modulation of humoral immune responses, while elevated *NOD1* expression in the liver and muscle was consistent with altered expression of intracellular pathogen-sensing mechanisms (95, 96). However, because the symptomatic fish were collected from the outbreak site and the apparently healthy comparison fish were obtained from separate culture facilities, environmental and physiological differences between the culture systems cannot be excluded as contributors to the observed transcriptional variation. Therefore, the present RT-qPCR findings should be interpreted as disease-associated immune transcriptional changes rather than direct evidence of *E. gallinarum*-specific immune modulation. Similarly, the observed reduction in *CR, TLR4* and *MHC II* transcripts may reflect altered immune-recognition and antigen-presentation processes during the disease condition, but does not by itself demonstrate direct bacterial interference with these pathways or pathogen-mediated immune evasion (20). Controlled infection experiments incorporating pathogen-confirmed infected and uninfected fish, together with defined post-infection sampling, will be required to establish the mechanistic effects of *E. gallinarum* on host immune pathways.

## Conclusion

The present study provides an integrated characterization of a field-recovered *Enterococcus gallinarum* isolate associated with disease and mass mortality in *Hypophthalmichthys nobilis*. The combination of field association, experimental challenge, bacterial re-isolation, multi-organ histopathology and tissue-specific host transcriptional responses provides complementary evidence for the pathogenic potential of the isolate under experimental conditions. The field investigation identified severe clinical and pathological alterations in moribund fish, while environmental assessment revealed several potentially stressful water- and sediment-quality conditions. The isolate was characterized using biochemical and 16S rRNA gene-based approaches and subsequently demonstrated pathogenic potential in a controlled experimental challenge, in which exposure was associated with rapid and complete mortality compared with the PBS control. Notably, the *E. gallinarum* isolate investigated in the present study (strain IFEGHNEK1) was originally isolated from *H. nobilis* during a mortality event and has been described in two separate publications. Bag et al. (97) investigated the effect of repeated subculturing on the virulence characteristics of this isolate, whereas Das et al. (70) reported its draft/complete genome sequence as *E. gallinarum* IFEGHNEK1 isolated from bighead carp. This study builds on these earlier reports but addresses a distinct research question: the pathogenicity of *E. gallinarum* in its associated host, *H. nobilis*, and the resulting pathological and host immune responses. The isolate exhibited a multidrug-resistant phenotype with a high multiple antibiotic resistance (MAR) index, indicating its adaptation to an environment subjected to considerable antibiotic selection pressure. Furthermore, the bacterium demonstrated strong virulence attributes, including enhanced biofilm formation, swimming motility, and β-hemolytic activity, suggesting a high capacity for host colonization, persistence, and tissue invasion. Histopathological examination revealed severe pathological alterations in the liver, kidney, muscle, and spleen of infected fish, confirming the systemic nature of the infection. In parallel, transcriptional analysis of immune-related genes revealed pronounced tissue-specific modulation of stress-, inflammatory-, and immune-related transcripts. The upregulation of *Hsp70, C3, NF-κB, IgM*, and *NOD1* in selected tissues was consistent with activation of stress and immune-associated responses, whereas the suppression of CR, TLR4, and MHC II in others indicated altered immune-related transcription during the disease condition. Because the immune-expression comparison involved field-collected symptomatic fish and apparently healthy fish from separate culture facilities, these transcriptional differences cannot be attributed exclusively to *E. gallinarum* infection. Further controlled infection studies with defined post-infection sampling are required to determine whether these changes represent direct pathogen-mediated modulation of host immune pathways. These findings collectively demonstrate that *E. gallinarum* induces severe pathological damage while eliciting a complex host immune response that is insufficient to prevent disease progression under outbreak conditions. The findings highlight the growing significance of *E. gallinarum* as an opportunistic fish pathogen and emphasize the need for continuous disease surveillance, prudent antibiotic stewardship, improved biosecurity practices, and the development of alternative preventive strategies to mitigate bacterial disease outbreaks and ensure sustainable freshwater aquaculture production (27).

## Data Availability

The datasets generated and/or analyzed during the current study are available in the NCBI repository, accession numbers OR999570. The whole-genome sequence of IFEGHNEK1 is available in GenBank under accession number CP169314. The BioSample and BioProject accession numbers are SAMN43379292 and PRJNA1152912, respectively. The raw sequence data have been deposited in the Sequence Read Archive under accession number SRR30899084 (70).
