# Supplementary material for: Unraveling the virulence and host immune responses to Enterococcus gallinarum during a mass mortality event in bighead carp (Hypophthalmichthys nobilis)
Source: Front Immunol. 2026 Oct 27;17:1938548. doi: 10.3389/fimmu.2026.1938548 (PMC13747786; doi:10.3389/fimmu.2026.1938548)
Supplement: Supplementary file 1 [file DataSheet1.docx]

**Supplementary Table 1**. Inhibition-zone diameters (mm± SE) and corresponding susceptibility interpretations of *Enterococcus gallinarum* isolated from *Hypophthalmichthys nobilis*.

| **Antibiotics** | *Enterococcus gallinarum*  (mm± SE) |
| --- | --- |
| **NIT** | 22.31±3.08 |
| **NET** | 19±3.78 |
| **AMP** | 22±2.18 |
| **CPM** | 27.4±2.57 |
| **TE** | 8±1.08 |
| **DO** | 1.04±0.06 |
| **CAZ** | 24.03±2.74 |
| **E** | 1.1±0.04 |
| **CIP** | 31.32±2.8 |
| **OF** | 28.1±3.1 |
| **DC** | 5.4±1.06 |
| **NA** | 19.24±1.56 |
| **GEN** | 26.19±2.8 |
| **AMX** | 21.15±1.9 |
| **PB** | 3.15±1.03 |
| **CL** | 7±1.22 |
| **K** | 19±2.7 |
| **TOB** | 24±3.01 |
| **S** | 20.08±2.78 |
| **RIF** | 3.1±0.25 |
| **TR** | 24±3.24 |
| **PIT** | 20±1.6 |
| **C** | 22.82±1.39 |
| **CFM** | 19.16±2.68 |
| **FO** | 23.34±1.98 |
| **IPM** | 2.11±0.2 |
